# SNAP-25, a Known Presynaptic Protein with Emerging Postsynaptic Functions

**DOI:** 10.3389/fnsyn.2016.00007

**Published:** 2016-03-24

**Authors:** Flavia Antonucci, Irene Corradini, Giuliana Fossati, Romana Tomasoni, Elisabetta Menna, Michela Matteoli

**Affiliations:** ^1^Department of Medical Biotechnology and Translational Medicine, Università degli Studi di MilanoMilan, Italy; ^2^Istituto di Neuroscienze, Centro Nazionale RicercheMilan, Italy; ^3^Humanitas Clinical and Research Center, IRCCS RozzanoRozzano, Italy

**Keywords:** SNAP-25, synaptopathies, presynaptic role, postsynaptic role, brain diseases

## Abstract

A hallmark of synaptic specializations is their dependence on highly organized complexes of proteins that interact with each other. The loss or modification of key synaptic proteins directly affects the properties of such networks, ultimately impacting synaptic function. SNAP-25 is a component of the SNARE complex, which is central to synaptic vesicle exocytosis, and, by directly interacting with different calcium channels subunits, it negatively modulates neuronal voltage-gated calcium channels, thus regulating intracellular calcium dynamics. The SNAP-25 gene has been associated with distinct brain diseases, including Attention Deficit Hyperactivity Disorder (ADHD), schizophrenia and bipolar disorder, indicating that the protein may act as a shared biological substrate among different “synaptopathies”. The mechanisms by which alterations in SNAP-25 may concur to these psychiatric diseases are still undefined, although alterations in neurotransmitter release have been indicated as potential causative processes. This review summarizes recent work showing that SNAP-25 not only controls exo/endocytic processes at the presynaptic terminal, but also regulates postsynaptic receptor trafficking, spine morphogenesis, and plasticity, thus opening the possibility that SNAP-25 defects may contribute to psychiatric diseases by impacting not only presynaptic but also postsynaptic functions.

SNAP-25 is a component of the SNARE protein complex, which is involved in the exocytotic release of neurotransmitters during synaptic transmission. Through the coiled-coil assembly with syntaxin-1 and synaptobrevin, SNAP-25 mediates synaptic vesicle apposition to the presynaptic membrane permitting their Ca^2+^ triggered fusion. Consistently, the genetic ablation of this protein results in a complete block of synaptic transmission. SNAP-25 is present in two isoforms, a and b, resulting from alternative splicing of the exon 5 of the *Snap-25* gene, which are differentially expressed during development. SNAP-25a is expressed at the embryonic stage, while SNAP-25b becomes the major isoform during postnatal life (Bark, 1993; Bark and Wilson, 1994; Bark et al., 1995), a developmental trend which has been confirmed in humans (Prescott and Chamberlain, 2011).

In line with its central role in neuronal function, the *Snap-25* gene has been associated with several human neurological syndromes, including attention-deficit/hyperactivity disorder (ADHD), schizophrenia (Barr et al., 2000; Brophy et al., 2002; Kustanovich et al., 2003), and bipolar disorder (Etain et al., 2010). The protein appears therefore to represent a shared biological element among different psychiatric diseases.

Recently, several groups started to investigate the cellular and molecular mechanisms underpinning the SNAP-25 contribution to the onset of such pathologies, or, more likely, to the manifestations of specific traits typical of these diseases. A challenging scenario is now emerging, i.e., that some of the defects in diseases involving SNAP-25 might not exclusively result from the presynaptic role of the protein. Indeed, initially recognized as a presynaptic SNARE protein, the protein has been later shown to play additional non-SNARE roles and, very recently, even postsynaptic functions. The results of these lines of research are summarized in this review (see Table 1A).

Table 1**(A) Functions of SNAP-25 protein, (B) *Snap-25* polymorphisms discussed along the text**.

**Table:** 

**(A) SNAP-25 known function**	***In vitro***	***Ex vivo***	***In vivo***	**Human**	**References**
Neurotransmitter release	•	•	•	•	Oyler et al., 1989; Söllner et al., 1993a,b; Chapman et al., 1994; Poirier et al., 1998; Raciborska et al., 1998; Sutton et al., 1998; Washbourne et al., 2002; Sørensen et al., 2003; Jeans et al., 2007; Mohrmann et al., 2010; Shen et al., 2014
Modulation of VGCCs	•	•			Bennett et al., 1992; Yoshida et al., 1992; Lévêque et al., 1994; Martin-Moutot et al., 1996; Rettig et al., 1996; Zhong et al., 1999; Jarvis and Zamponi, 2001; Verderio et al., 2004; Pozzi et al., 2008; Condliffe et al., 2010; Condliffe and Matteoli, 2011; Weiss et al., 2012
Slow, clathrin-dependent endocytosis	•				Okamoto et al., 1999; Xu et al., 2013; Zhang et al., 2013
Postsynaptic receptor trafficking	•	•			Selak et al., 2009; Lau et al., 2010; Jurado et al., 2013
Short term plasticity	•	•			Pozzi et al., 2008; Antonucci et al., 2013
Long term plasticity	•	•			Jurado et al., 2013; Fossati et al., 2015
Dendritic spine morphogenesis	•		•		Tomasoni et al., 2013; Fossati et al., 2015
Cognitive ability, learning, and memory			•	•	Gosso et al., 2006, 2008; Corradini et al., 2014; Braida et al., 2015
Network excitability and epileptiform activity	•	•	•	•	Hess et al., 1992, 1995; Zhang et al., 2004; Rohena et al., 2013; Corradini et al., 2014; Shen et al., 2014

**Table:** 

**(B) Polymorphysm**	**Position in the gene**	**Traits**	**Effects on mRNA/protein**	**References**
rs6039769	Promoter	Early onset bipolar disorder	Higher SNAP-25 levels in homozygous “CC” individuals	Etain et al., 2010
rs363039	Intron 1	Association with variation in IQ in normal population; verbal performances in women; working memory capacity; cognitive traits in autistic children	Transcription binding site	Gosso et al., 2006; Cagliani et al., 2012; Söderqvist et al., 2010; Braida et al., 2015
rs363050	Intron 1	Association with variation in IQ in normal population; association with intellectual disabilities; association with Alzheimer's disease and mild cognitive impairment; cognitive traits in autistic children	Transcription binding site; reduced protein expression	Gosso et al., 2006; Rizzi et al., 2012; Guerini et al., 2014; Braida et al., 2015
rs363043	Intron 1	Association with variation in IQ in normal population; hyperactivity in autistic children; association with Alzheimer's disease and mild cognitive impairment;	Transcription binding site	Gosso et al., 2008; Guerini et al., 2011, 2014
rs353016	Intron 1	Association with variation in IQ in normal population	Transcription binding site	Gosso et al., 2008
rs6108461	Intron 3	ADHD—regulation of attention and inhibition	Decreased expression of SNAP-25	Hawi et al., 2013
rs362549	Intron 4	ADHD—inattentive trait, hyperactivity trait		Zhang et al., 2011
rs362990	Intron 4	ADHD—regulation of attention and inhibition	Decreased expression of SNAP-25	Hawi et al., 2013
rs363006	Intron 7	Early onset bipolar disorder; ADHD	N/D	Etain et al., 2010; Zhang et al., 2011
rs3746544	3′untranslated	ADHD traits, especially when associated to norepinephrine transporter NET1 (rs2242447); increased risk of schizophrenia and major depressive disorder	N/D	Carroll et al., 2009; Pazvantoğlu et al., 2013; Dai et al., 2014; Wang et al., 2015
rs1051312	3′untranslated	ADHD; cognitive dysfunction in schizophrenia; impultivity trait in healthy population when in haplotype with rs3746544	N/D	Brophy et al., 2002; Spellmann et al., 2008; Németh et al., 2013

*Only a selection of papers describing the role of SNAP-25 in the control of neurotransmitter release is reported owing to space limitations (see text for details). The position in the gene, traits associated with the genetic variant and effect on mRNA or protein levels are listed*.

## Role of SNAP25 at the presynapse: synaptic vesicles exocytosis and short term plasticity

SNAP-25 (synaptosomal-associated protein of 25 kDa) is a soluble N-ethylmaleimide sensitive factor attachment protein receptor (SNARE) protein that participates together with syntaxin-1 and synaptobrevin/VAMP (Jahn et al., 2003; Sudhof, 2004; Montecucco et al., 2005) in the regulation of synaptic vesicle exocytosis (Washbourne et al., 2002; reviewed in Milovanovic and Jahn, 2015). In the absence of SNAP-25, vesicle docking at the presynaptic active zones persists, but the pool of vesicles primed for release is empty, and fast calcium-triggered exocytosis is abolished (Sørensen et al., 2003). Furthermore, by calcium-dependent interaction with synaptotagmin, SNAP25 has a role in vesicle docking and priming as well as in triggering fast exocytosis (Mohrmann et al., 2010). Indeed the proteolytic cleavage of SNAP-25 by botulinum neurotoxins (BoNTs, serotypes A, C, and E) blocks exocytosis and neurotransmitter release (Schiavo et al., 2000; Ahnert-Hilger et al., 2013; Pantano and Montecucco, 2014), leading to the neuroparalysis characteristic of botulism (Aoki and Guyer, 2001).

**Figure 1:**
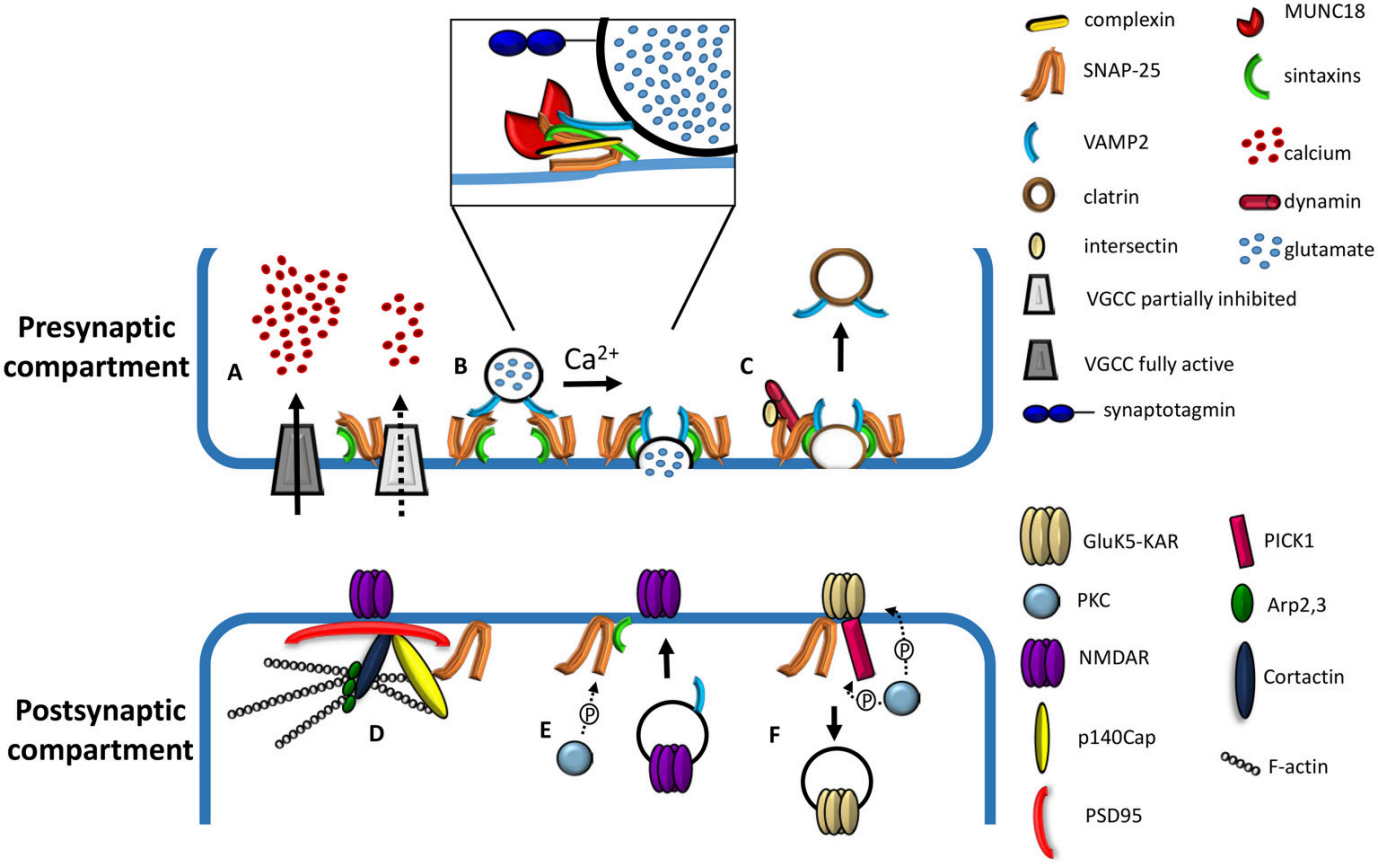
**Cartoon depicting presynaptic and postsynaptic roles of SNAP-25. (A)** Effect of presynaptic SNAP-25 on VGCCs. Calcium influx in the nerve terminal is negatively regulated by the complex formation between SNAP-25 and VGCCs; removal of the clamping role of SNAP-25, occurring upon reduction of the protein expression, results in elevated calcium influx through VGCCs (adapted from Kochlamazashvili and Haucke, 2013). **(B)** Involvement of SNAP-25 in the molecular machinery mediating Ca^2+^-triggered vesicle fusion. A docked synaptic vesicle is shown on the left. The core fusion machine is composed of synaptobrevin/VAMP2, syntaxin-1, and SNAP-25 (adapted from Kochlamazashvili and Haucke, 2013). The diagram in the box depicts a partially assembled SNARE complex including, besides synaptobrevin/VAMP2, syntaxin-1, and SNAP-25, complexins and MUNC18. The calcium sensor, synaptotagmin, is also depicted (adapted from Sudhof, 2012). **(C)** Role of SNAP-25 in slow clathrin-mediated synaptic vesicle endocytosis. SNAP-25 binds to the endocytic protein intersectin, while syntaxin binds dynamin, a GTPase mediating vesicle fission. The interactions between Synaptobrevin/VAMP2 and the ANTH domain of endocytic adaptors AP180 and CALM have been omitted for clarity. **(D)** Role of SNAP-25 in the organization of the postsynaptic density protein network. SNAP-25 interacts with p140Cap, which in turn forms a complex with PSD95, cortactin, Arp2,3, and F-actin (filamentous actin). NMDA receptors are depicted as interacting with PSD95 (adapted from Fossati et al., 2015). **(E)** Phosphorylation of SNAP-25 by PKC promotes the insertion of NMDA channels at the cell surface through the delivery of postsynaptic vesicles and their fusion with the plasma membrane, possibly via the formation of a SNARE complex (adapted from Lau et al., 2010). **(F)** Role of SNAP-25 in the removal of GluK5-contaning kainate receptors (KAR). KARs associate with SNAP-25 and the PKC-interacting protein PICK1. The PKC phosphorylation of the GluK5-C terminus may induce a conformational change facilitating the association with SNAP-25 and simultaneously decreasing GRIP binding affinity (adapted from Selak et al., 2009).

Besides its well characterized role in exocytosis, SNAP-25 also modulates various voltage-gated calcium channels (VGCCs) (Atlas et al., 2001; Zamponi, 2003; Catterall and Few, 2008), by interacting with N-type (Sheng et al., 1996), P/Q-type (Martin-Moutot et al., 1996; Rettig et al., 1996), L-type (Wiser et al., 1999), and T-type channels (Weiss et al., 2012). SNAP-25 has been shown to negatively control neuronal calcium responsiveness to depolarization (Verderio et al., 2004) through voltage-gated calcium channel inhibition (Pozzi et al., 2008). Consistently, silencing endogenous SNAP-25 in glutamatergic neurons results in increased VGCC activity (Condliffe et al., 2010; Condliffe and Matteoli, 2011; see Figure 1).

SNAP-25 also participates in slow, clathrin-dependent endocytosis at hippocampal synapses, possibly contributing to the coupling between exocytosis and endocytosis (Zhang et al., 2013). Given that SNARE proteins mediate exocytosis at all nerve terminals, their dual role in exo- and endocytosis is likely a general principle. Although how exactly SNARE proteins are involved in endocytosis remains unclear, the following binding studies provide some indications. Synaptobrevin/VAMP2 binds to the AP180 N-terminal homology (ANTH) domain of endocytic adaptors AP180 and Clathrin Assembly Lymphoid Myeloid leukemia (CALM) protein (Koo et al., 2011; Miller et al., 2011); also, stonin 2, facilitates clathrin/AP-2-dependent internalization of synaptotagmin and targets it to a recycling vesicle pool in living neurons (Diril et al., 2006).

SNAP-25 binds to the endocytic protein intersectin (Okamoto et al., 1999); syntaxin binds to dynamin (Galas et al., 2000). Based on these evidence it was proposed that the exocytosis machinery, including SNARE proteins (synaptobrevin, SNAP-25, and syntaxin), is needed in the initiating step of endocytosis and likely controls the amount of endocytosis (for a review see Wu et al., 2014).

Therefore, SNAP-25 represents a multifunctional protein involved in the control of secretion by multiple interactions. In line with the multiple roles of the protein, different neuronal processes are affected, in an unexpected way, in conditions characterized by SNAP-25 reduction. Indeed halved SNAP-25 levels in 13–14 DIV neuronal cultures not only failed to impair synaptic transmission, as expected by the SNARE role of SNAP-25, but instead enhanced evoked glutamatergic neurotransmission (Antonucci et al., 2013). This effect was dependent on presynaptic voltage-gated calcium channel activity and was not accompanied by changes in spontaneous quantal events or in the pool of readily releasable synaptic vesicles (Antonucci et al., 2013). Notably, synapses of 13–14 DIV neurons with reduced SNAP-25 expression showed paired-pulse depression as opposed to paired-pulse facilitation occurring in their wild-type counterparts (Antonucci et al., 2013). These data suggest that the more sensitive phenotype for reduced SNAP-25 levels may be the regulation of calcium channels, not the role of SNAP-25 in transmitter release. Based on these results, a dual role of SNAP-25 not only as a carrier but also as a “guardian of synaptic transmission” was proposed: in particular, reduced SNAP-25 expression, although sufficient to sustain SNARE-mediated synaptic vesicle fusion, partially releases VGCCs from SNAP-25-mediated inhibition, thus resulting in elevated calcium influx and facilitated neurotransmission (Kochlamazashvili and Haucke, 2013).

## An unexpected role of SNAP-25 at the post-synapse: spine morphogenesis and plasticity

In the last years, different evidence indicated an unexpected postsynaptic role for SNAP-25 (see Figure 1). The protein was indeed shown to control NMDA and kainate-type receptors trafficking (Selak et al., 2009; Lau et al., 2010). In particular the interaction of SNAP-25 with the GluK5 subunit of KARs and PICK1 reduces the GluK5 stability on the membrane, thus favoring KAR internalization (Selak et al., 2009), whereas the PKC-mediated phosphorylation of SNAP-25 on serine 187, promotes NMDAR delivery to the cell surface via SNARE-dependent exocytosis (Lau et al., 2010). In the latter study the authors elegantly demonstrated that introduction of the constitutively active form of PKC via the recording pipette to neurons rapidly potentiated NMDA currents in cells treated with inactive BoNT/A whereas treatment of neurons with active BoNT/A abolished PKC potentiation of NMDA currents without altering basal NMDA currents, thus unveiling SNAP-25 involvement in the potentiation of the synapse. Given that LTP-inducing protocols can induce SNAP-25 phosphorylation (Genoud et al., 1999), high frequency stimulation protocols may act via phosphorylation of SNAP-25 to promote insertion of NMDARs and elicit LTP. Indeed acute SNAP-25 downregulation resulted in LTP impairment (Jurado et al., 2013). These data opened the possibility that, besides a presynaptic impact, reductions of SNAP-25 levels may affect the structure, and/or the function of the postsynaptic compartment, which would provide a logical frame for the protein involvement in psychiatric diseases, such as schizophrenia or intellectual disability, which are known to be also characterized by defects at the postsynaptic compartment (Fernández et al., 2009; Penzes et al., 2011).

Despite the evidence pointing to a postsynaptic role of SNAP-25, a clear demonstration of whether SNAP-25 localizes in the dendritic spines of the postsynaptic neuron is still lacking. Some recent studies attempted to locate SNAP-25 in the postsynaptic terminal either by immunofluorescence (Selak et al., 2009), or ground state depletion (GSD) microscopy, which allows protein localization with a precision up to 20 nm (Tomasoni et al., 2013). Also by coimmunoprecipitation, bimolecular fluorescence complementation (BiFC) and biochemical fractionation, a molecular complex of SNAP-25 with postsynaptic proteins was detected (Selak et al., 2009; Tomasoni et al., 2013; Fossati et al., 2015). Nevertheless this is still a controversial topic, since other studies showed an exclusively presynaptic location of SNAP-25 through immunogold labeling of synaptic boutons (Holderith et al., 2012; Kerti et al., 2012). Certainly, the SNAP-25 expression levels in the postsynaptic compartment are quantitatively much lower than at the presynaptic one (Tao-Cheng et al., 2000; Sharma et al., 2012) and this could account for its difficult detection in dendritic spines.

In recent years, the postsynaptic role of SNAP-25 has been supported by evidence showing a structural modification of the postsynaptic compartment upon SNAP-25 reduction. In particular, acute reduction of SNAP-25 expression in primary hippocampal cultures led to an immature phenotype of dendritic spines, while overexpression of the protein resulted in an increase in the density of mature, PSD-95-positive spines (Tomasoni et al., 2013). The effect was shown to be truly postsynaptic, and not secondary to altered presynaptic function as demonstrated by co-culturing of SNAP25 heterozygous and GFP-expressing wild type neurons. SNAP-25 reductions were also shown to affect the localization of PSD95, with acute downregulation of SNAP-25 resulting in a significant reduction of PSD95-positive puncta (Fossati et al., 2015). Correspondingly, acute down-regulation of SNAP-25 in CA1 hippocampal region by lentiviral expression reduced spine density and resulted in immature spine morphology, thus recapitulating *in vivo* the spine abnormalities observed in cultures upon acute SNAP-25 silencing (Fossati et al., 2015).

Which could be the mechanism by which SNAP-25 controls dendritic spine morphology and PSD95 mobility? The cleavage of SNAP-25 by BoNT/E, which prevents the protein to enter the fusion complex, did not reduce spine density or PSD95 size, thus excluding that SNAP-25 controls PSD95 recruitment through its SNARE function and suggesting instead a protein scaffolding role at the spine level (Fossati et al., 2015). This hypothesis was supported by the finding that p140Cap, a scaffold protein located into dendritic spines with a crucial role in regulating actin cytoskeleton, spine formation (Jaworski et al., 2009), and learning processes (Repetto et al., 2014), is a key member of the molecular complex which includes SNAP-25 and PSD95 (Tomasoni et al., 2013; Fossati et al., 2015).

The correct formation of this molecular complex preserves the proper organization of the dendritic spine. Maintaining spine integrity could further facilitate the formation of the protein complexes which contain also SNAP-25 and that regulate receptor trafficking (Selak et al., 2009; Lau et al., 2010). Based on these results, it is conceivable that postsynaptic SNAP-25 may be important for orchestrating a dynamic equilibrium among the glutamate receptors at a given synapse, thereby regulating synapse efficacy also at the postsynaptic side.

## SNAP-25, a shared biological pathway among different psychiatric diseases

The defective formation of the SNARE complex for vesicle fusion and the aberrant regulation of voltage-gated calcium channels are the processes generally taken into account to explain the involvement of the protein in those psychiatric diseases which have been linked to the *Snap-25* gene. However, the recent data indicating a postsynaptic role for the protein raise the possibility that SNAP-25 defects may contribute, in these disorders, also through alterations of postsynaptic receptors trafficking or spine morphogenesis.

Several reports have shown the presence of polymorphisms in the *Snap-25* gene, which have been associated with ADHD (Barr et al., 2000; Faraone et al., 2005; Zhang et al., 2011; Hawi et al., 2013; Pazvantoğlu et al., 2013), schizophrenia (Thompson et al., 2003), and early-onset bipolar disorders (Etain et al., 2010; see Table 1B). Notably, some of these polymorphisms were found to control not only specific traits of the disease, but even behavioral tracts in healthy individuals. As an example, several single nucleotide polymorphisms (i.e., rs363043, rs353016, rs363039, rs363050) of the *Snap-25* gene have been associated with Intelligence Quotient (IQ) phenotypes in healthy individuals (Gosso et al., 2006, 2008). Also, although autism spectrum disorder (ASD) has not been directly linked to the *Snap-25* gene, polymorphisms analyzed in a cohort of children affected by ASD revealed a significant association between *Snap-25* SNP rs363043 and hyperactivity traits (Guerini et al., 2011), while rs363050 and rs363039 polymorphisms were shown to correlate with cognitive deficits in ASD children (Braida et al., 2015). Notably, a first analysis of transcriptional activity through luciferase reporter gene revealed that SNP rs363050, which is localized in the intron 1 of the *Snap-25* gene, leads to reduced protein expression (Braida et al., 2015). Therefore, the possibility that reduced SNAP-25 levels may contribute to specific behavioral traits, such as hyperactivity or cognitive performances in healthy individuals or in different psychiatric diseases, including those to which the gene has not been directly associated, like in the case of ASD, remains a challenging possibility to be tested in the future.

Notably, even in schizophrenia, where the SNAP-25 levels are significantly lower in the hippocampus (Young et al., 1998; Fatemi et al., 2001; Thompson et al., 2003) and in the frontal lobe Broadman's area 10 (Thompson et al., 1998), an association between the rs1051312 polymorphism of the *Snap-25* gene and cognitive dysfunctions was reported (Spellmann et al., 2008). Furthermore, and consistent with the observations already reported in SNAP-25 heterozigous mice (Antonucci et al., 2013), even in schizophrenic patients the reduction of SNAP-25 levels does not seem to correlate with an impairment in the SNARE complex formation (Ramos-Miguel et al., 2015). Of interest, and in line with the possible relevance of SNAP-25 expression levels in different psychiatric diseases, a SNAP-25 variant located in the promoter region (rs6039769) and associated with early-onset bipolar disorder was found to correlate with a significantly higher SNAP-25b expression in prefrontal cortex (Etain et al., 2010). Higher levels of the SNARE in dorsolateral prefrontal cortex of patients affected by bipolar disorder were already reported by Scarr et al. (2006).

As a support to the functional impact of the protein levels in cognitive or motor functions, genetic mice models showed that the chronic reduction of SNAP-25 affects mouse behavior. The coloboma mouse model, characterized by halved SNAP-25 levels (Hess et al., 1992), displays indeed a hyperactive phenotype (Hess et al., 1992), associated with abnormal thalamic spike-wave discharges (Hess et al., 1995; Zhang et al., 2004; Faraone et al., 2005; Russell, 2007). Similarly, juvenile SNAP-25 heterozygous mice displays a moderate hyperactivity, which disappears in the adult animals, and impaired associative learning and memory, which persist instead in the adults. Electroencephalographic recordings revealed the occurrence of frequent spikes, suggesting a diffuse network hyperexcitability, accompanied by a higher susceptibility to kainate-induced seizures, and degeneration of hilar neurons. Notably, both EEG alterations and cognitive defects were improved by antiepileptic drugs, in particular valproic acid (Corradini et al., 2014; Braida et al., 2015). A defective negative control of voltage gated calcium channels resulting from the reduced SNAP-25 levels could be at the origin of the network hyperexcitability (Corradini et al., 2014). Although, the demonstration of a direct causal link between altered SNAP-25 expression and psychiatric diseases is still lacking, evidences obtained in the coloboma mouse suggest that reduction of SNAP-25 expression may be directly involved in some psychiatric traits, rather than simply represent an epiphenomenon; indeed, when a transgene expressing SNAP-25 was bred into the coloboma strain in order to complement Snap-25 depletion, the hyperactivity displayed by the mutant mice was rescued (Hess et al., 1995 J Neurosci).

Recently a *de novo* variant was identified in the *Snap-25* exon 4 (Phe48Val), in a 15-years-old girl with intractable epilepsy and severe encephalopathy, but no neuromuscular symptoms (Rohena et al., 2013). Later on, exome sequencing identified a *de novo* dominant mutation of a conserved residue in exon 5 of *Snap-25b* in an 11-years-old patient displaying congenital myasthenia, cortical hyperexcitability, cerebellar ataxia, and intellectual disability (Shen et al., 2014). The Ile67Asn variant was reported to be pathogenic because, by disrupting the hydrophobic alpha-helical coiled-coil structure of the SNARE complex, it inhibits synaptic vesicle exocytosis (Shen et al., 2014). Of note, a heterozygous Ile67Thr missense mutation in *Snap-25b* gene was observed in the so-called blind-drunk (1/Bdr) mouse, which shows a mild ataxic gait around age 4 weeks, impaired sensorimotor gating and increased anxiety (Jeans et al., 2007; Oliver and Davies, 2009). This mutation is located in a highly conserved codon and parallels Ile67Asn mutation observed in the 11 years-old patient (Shen et al., 2014). In the case of the Ile67Asn mutation, Shen and colleagues propose that the substitution of a hydrophobic residue with a hydrophilic one destabilizes the coiled-coil SNARE complex structure, thus hindering vesicle fusion (Shen et al., 2014); however, it is also possible that the Ile67Asn mutation causes a distortion of the coiled coil structure in such a way as to affect the interaction of the SNARE complex with its protein partners. This appears to be the case in the blind-drunk mutation which results in the enhancement of the affinity of SNAP-25 for its binding partners and is therefore likely to cause an increase in association of the SNARE complex (Jeans et al., 2007). No information about the impact of Val48Phe variant on SNAP-25 structure and function is still available.

Additional genetic mouse models underlined the role of *Snap-25* mutations in specific traits of psychiatric diseases. Single nucleotide substitution resulting in a missense Ser187Ala mutation at the site of phosphorylation of SNAP-25 by PKC has been associated with increased anxiety, decreased dopamine and serotonine release (Kataoka et al., 2011), impaired PPI of the startle response, a typical parameter of schizophrenia, deficits in working memory, immature features of dentate granule cells (Ohira et al., 2013), and epileptic seizures (Watanabe et al., 2015). Interestingly, Ser187 phosphorylation of SNAP-25 is development- and activity-dependent both *in vitro* and *in vivo* (Kataoka et al., 2006; Pozzi et al., 2008); it is associated with synaptic vesicles availability (Nagy et al., 2002; Houeland et al., 2007) and it is necessary for the negative control of voltage-gated calcium channels (Pozzi et al., 2008).

## Conclusions

The recent discovery of SNAP-25 role in the control of receptor trafficking and spine morphogenesis, which points to the protein role as a postsynaptic structural hub, opens new avenues for the comprehension of the physiological role of the protein at the synapse and offers new mechanistic insights as to SNAP-25 involvement in synaptopathies that go beyond the protein's established roles in presynaptic function. The finding that the activity-driven spine remodeling is defective in neuronal networks constitutively developing in the presence of reduced levels of SNAP-25, makes a provocative link to human pathologies, such as schizophrenia, where both a reduction of SNAP-25 expression and a decrease in dendritic spine density have been described.

## Author contributions

All authors listed, have made substantial, direct and intellectual contribution to the work, and approved it for publication.

### Conflict of interest statement

The authors declare that the research was conducted in the absence of any commercial or financial relationships that could be construed as a potential conflict of interest.
